# Transcriptome-wide Profiling of Cerebral Cavernous Malformations Patients Reveal Important Long noncoding RNA molecular signatures

**DOI:** 10.1038/s41598-019-54845-0

**Published:** 2019-12-03

**Authors:** Santhilal Subhash, Norman Kalmbach, Florian Wegner, Susanne Petri, Torsten Glomb, Oliver Dittrich-Breiholz, Caiquan Huang, Kiran Kumar Bali, Wolfram S. Kunz, Amir Samii, Helmut Bertalanffy, Chandrasekhar Kanduri, Souvik Kar

**Affiliations:** 1 International Neuroscience Institute, Rudolf-Pichlmayr-Strasse 4, D-30625 Hannover, Germany; 2 Department of Medical Biochemistry and Cell Biology, Institute of Biomedicine, Sahlgrenska Academy, University of Gothenburg, Gothenburg, 40530 Sweden; 3 Present Address: Neopep Pharma GmbH & Co. KG, Feodor Lynen Strasse 31, 30625 Hannover, Germany; 4 Department of Neurology, Hannover Medical School, Hannover, Germany; 5 Research Core Unit Genomics, Hannover Medical School, Hannover, Germany; 6 Department for Experimental Pain Research, Center of Biomedicine and Medical Technology Mannheim (CBTM), Medical Faculty Mannheim, Heidelberg University, Mannheim, Germany; 7 Institute of Experimental Epileptology and Cognition Research and Department of Epileptology, Life and Brain Center, University Hospital Bonn, Sigmund-Freud-Strasse 25, D-53105 Bonn, Germany

**Keywords:** Long non-coding RNAs, Transcriptomics, Diseases, Computational biology and bioinformatics

## Abstract

Cerebral cavernous malformations (CCMs) are low-flow vascular malformations in the brain associated with recurrent hemorrhage and seizures. The current treatment of CCMs relies solely on surgical intervention. Henceforth, alternative non-invasive therapies are urgently needed to help prevent subsequent hemorrhagic episodes. Long non-coding RNAs (lncRNAs) belong to the class of non-coding RNAs and are known to regulate gene transcription and involved in chromatin remodeling via various mechanism. Despite accumulating evidence demonstrating the role of lncRNAs in cerebrovascular disorders, their identification in CCMs pathology remains unknown. The objective of the current study was to identify lncRNAs associated with CCMs pathogenesis using patient cohorts having 10 CCM patients and 4 controls from brain. Executing next generation sequencing, we performed whole transcriptome sequencing (RNA-seq) analysis and identified 1,967 lncRNAs and 4,928 protein coding genes (PCGs) to be differentially expressed in CCMs patients. Among these, we selected top 6 differentially expressed lncRNAs each having significant correlative expression with more than 100 differentially expressed PCGs. The differential expression status of the top lncRNAs, *SMIM25* and *LBX2-AS1* in CCMs was further confirmed by qRT-PCR analysis. Additionally, gene set enrichment analysis of correlated PCGs revealed critical pathways related to vascular signaling and important biological processes relevant to CCMs pathophysiology. Here, by transcriptome-wide approach we demonstrate that lncRNAs are prevalent in CCMs disease and are likely to play critical roles in regulating important signaling pathways involved in the disease progression. We believe, that detailed future investigations on this set of identified lncRNAs can provide useful insights into the biology and, ultimately, contribute in preventing this debilitating disease.

## Introduction

Cerebral cavernous malformations (CCMs) are vascular lesions of the brain affecting approximately 0.5% of the human population^1,2^. They are associated with leaky endothelium and increased vascular permeability which often results in seizures, intracerebral hemorrhage and focal neurological deficits. CCMs can occur either sporadically with single lesion or as familial forms harboring multiple lesions. Familial CCMs are inherited in an autosomal dominant pattern with incomplete penetrance and variable expressivity^3,4^. Loss-of-function mutations are known in one of the three CCM genes: Krev interaction trapped 1 (*CCM1*/*KRIT1*), cerebral cavernous malformations 2 (*CCM2*) and programmed cell death 10 (*PDCD10*) predisposes to CCMs^5,6^. However, a growing body of evidence suggests that mutations of CCM genes are alone not fully sufficient to induce CCM lesion burden, therefore indicating the involvement of yet to be identified genetic factors for the disease progression^7^.

Noncoding RNAs represent RNA molecules that are not translated into proteins and are known to be involved in chromatin remodeling, post-transcriptional modifications, signal transduction and disease progression^8–10^. Based on their transcript size they are classified into two major categories: small non-coding RNAs of <200 nucleotides in length (microRNAs, snoRNAs, scaRNAs and piRNAs) and long non-coding RNAs (lncRNAs) of >200 nucleotides in length^11^. From our previous work, using RNA sequencing approach, we have shown that there are microRNAs and snoRNAs significantly deregulated in CCMs disease^1,3^. In this current study we focused on identifying and profiling long noncoding RNA (lncRNA) molecules in CCMs patients.

Long non-coding RNAs (lncRNAs) regulate gene expression by various mechanisms, such as chromatin level regulation, act as enhancer function, involvement in protein stability and modulation of transcription factor activity^8,9,12^. The role of lncRNAs in ischemic stroke and cerebrovascular pathologies has been well established^13,14^. Recently, Dykstra-Aiello *et al*. reported the correlation of lncRNAs identified in blood samples from stroke affected patients with the vascular risk factors^15^. Other studies too identified aberrantly expressed lncRNAs in focal ischemia, Hereditary Hemorrhagic Telangiectasia (HHT) and brain arteriovenous malformations (AVM) showing their clinical relevance in cerebrovascular malformations^16–18^. However, till date, the role of lncRNAs in CCMs disease has not been reported. Despite recent studies reporting the involvement of lncRNAs in animal models of stroke and cerebrovascular pathologies^13,16^, their role in CCMs disease remains elusive. We, therefore, investigated their association from surgically resected CCMs lesions. By using RNA-sequencing approach for profiling lncRNAs and their co-expressed protein coding genes (PCGs), this study will provide a new perspective on CCMs treatment strategies. Recent studies have investigated the functional relevance of lncRNAs using different computational approaches such as co-expression analysis and lncRNA-protein coding gene proximity analysis (cis or trans-regulation) strategies^8,19–21^. Since we used transcriptome or expression-based approach, we implemented lncRNA-mRNA co-expression analysis strategy to find functionally relevant lncRNAs deregulated in CCMs. Overall, this study is a unique effort to catalogue lncRNAs associated with CCMs disease and might open up new directions for better improved alternative therapies.

## Results

### Transcriptome profiling of CCM samples

CCMs originating from brainstem are rare and of particular interest due to their crucial relationship with the adjacent vascular and neural structures. Any mild or undetectable changes in these regions may result in higher bleeding rate associated with severe neurological deficits and morbidity^22^. Current treatment options for brainstem CCMs rely only on microsurgical interventions. Henceforth, alternative non-invasive treatment modalities are a prerequisite for preventing rebleeding in such eloquent locations.

**Figure 1:**
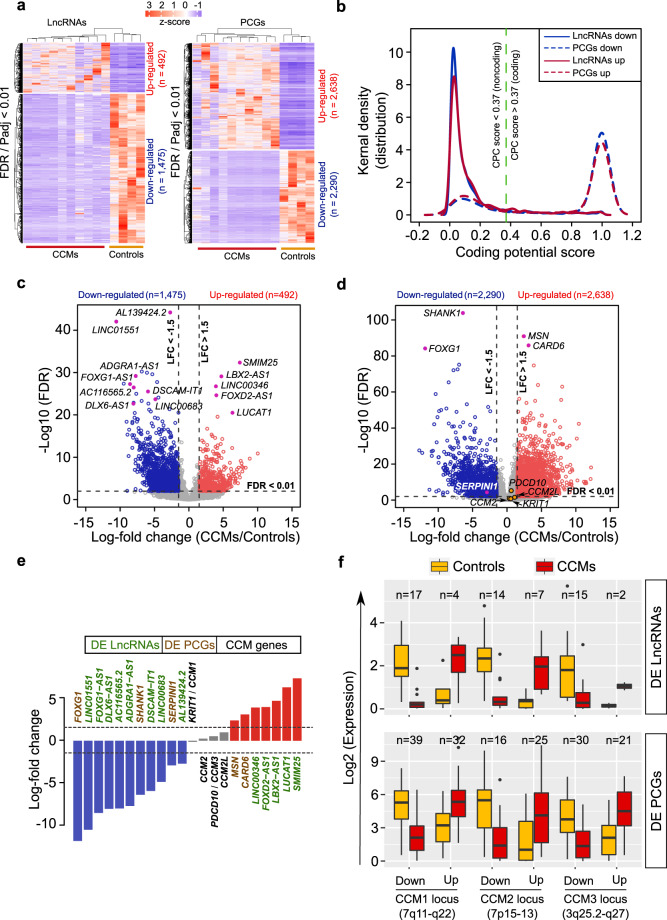
Transcriptome profiles of CCM patients with differential expression patterns of LncRNAs and PCGs. (**a**) Heatmap showing differential expression of lncRNAs and PCGs between CCMs (n = 10) and control group (n = 4). (**b**) Kernal density graph showing coding potential probability of DE lncRNAs and DE PCGs. Probability or score is calculated using coding potential calculator (CPC). Green dotted lines divide coding and noncoding CPC scores. (**c**,**d**) Volcano plots shows up- (red) and down-regulated (blue) lncRNAs (**c**) and PCGs (**d**) respectively. Key significantly differentially expressed transcripts are highlighted with pink and known CCM related PCGs are highlighted with yellow. Vertical dotted lines represent log-fold change cut-off ±1.5 (right and left) and values above horizontal dotted lines represent transcripts with FDR <0.05 cut-off. (**e**) Expression status of CCM genes (grey bar) along with top up- (red bar) and down-regulated (blue bar) DE lncRNAs and PCGs. (**f**) Boxplots showing differentially expressed transcripts (lncRNAs and PCGs) on three previously known CCM susceptibility locus.

Following total RNA isolation from 10 brainstem CCMs (Table 1) and 4 temporal lobe epilepsy (TLE) controls (Table 2), samples were processed for library preparation and RNA sequencing (Fig. 1a). After the FASTQ files were adapter and quality trimmed, 70 to 92 million read sequences belonging to each sample were mapped to the human genome hg38, available on illumina’s iGenome site (http://support.illumina.com/sequencing/sequencing_software/igenome.html). The average number of reads entering the mapping process across all analyzed samples was 85.7 million. The average percentage of uniquely mapped reads was 85.4%, of reads mapped to multiple loci was 12.5%, and of unmapped reads was 1.8%.

**Table 1:** CCMs patient clinical features.

CCM Patient Data													
Patient No.	Age/Sex	Clinical presentation	Seizures	Hemorrhagic episodes	Radiological findings	Family history	Lesion location	Size (Diameter) mm	Multiple lesions	DVA	Ethnicity	Edema	Radiation-induced
CCM 1	40/M	SH	N	2	NRH	N	R/Pontine	21	N	Y	1	Y(slight)	N
CCM 2	12/F	SH	N	1	NRH	N	R/Pontomesencephalic	42.3	N	N	3	N	Y
CCM 3	32/F	SH	N	3	NRH	N	R/Pontine	36	N	Y	1	Y	N
CCM 4	50/F	NH-FND	Y	2	NRH	N	R/Medulla oblongata	16	Y	N	1	N	Y
CCM 5	25/M	SH	N	1	RH	N	R/Pontine	25.2	N	Y	3	Y	N
CCM 6	40/F	SH	N	3	NRH	N	L/Pontine	13.4	N	N	1	N	N
CCM 7	19/F	SH	N	1	RH	N	R/Pontine	27	N	Y	3	Y	N
CCM 8	45/F	SH	N	1	NRH	N	R/Middle cerebellar ped	13	N	N	1	Y	N
CCM 9	30/M	SH	N	2	RH	N	L/Pontine	15	N	N	3	Y	N
CCM10	46/F	SH	N	1	NRH	N	L/Medulla oblongata	9,14	N	N	1	N	N

NRH, no recent hemorrhage; RH, recent hemorrhage; F, female; M, male; Y, yes; N, no; NH-FND, non-hemorrhagic focal neurological deficit; SH, symptomatic hemorrhage; Ethnicity: 1, White/European descent; 2, African; 3, Arabian; 4, Hispanic; 5, Asian.

**Table 2:** TLE control clinical information.

TLE Control Data				
Patient No.	Age/Sex	Location	Histological Diagnosis	Clinical diagnosis
Control1	18/F	Amygdala	Lesion	epilepsy with partial seizures after middle cerebral artery infarction
Control2	21/M	Amygdala	Lesion	epilepsy with complex focal seizures
Control3	30/M	Amygdala	Lesion	epilepsy associated with porencephaly in white matter and insula region right
Control4	24/M	Amygdala	Lesion	MRI negative focal epilepsy

TLE, temporal lobe epilepsy.

### Differentially expressed lncRNAs and protein coding genes (PCGs) in CCMs

By comparing expression patterns between CCMs (n = 10) and control groups (n = 4) from RNA-seq, differentially expressed (DE) up- and down-regulated lncRNAs and protein coding genes (PCGs) were profiled. Among them, 1,967 lncRNAs and 4,928 PCGs were significantly differentially expressed (DE) between CCMs and control comparison groups. There were 1,475 and 2,290 up-regulated lncRNAs and PCGs; 492 and 2,638 down-regulated lncRNAs and PCGs, respectively. These differentially expressed (DE) transcripts were filtered using corrected p-value (p value adj <0.01) and an absolute log-fold change greater than 1.5 between the comparison groups (Fig. 1a and Supplementary Data 1). These transcripts were then tested for their coding potential capacity using CPC (coding potential calculator) to ensure that the obtained DE lncRNAs do not code for any peptide. CPC prediction results showed no peptide coding capability for lncRNAs (CPC score less than 0.37 represents noncoding transcript) and the DE PCGs showed high coding capability. There are certain PCGs seen towards noncoding portion due to their noncoding transcript isoforms that are previously known to function as both coding and noncoding RNAs (cncRNAs or bi-functional RNAs)^23^ (Fig. 1b). Detailed investigation of the top DE transcripts revealed lncRNAs involved in CNS related disorders (*FOXG1-AS1* and *LINC01551*)^24,25^. In addition to the latter our analysis also revealed lncRNAs (*DLX6-AS1*) that regulate the expression of distant mRNAs (*DLX6-AS1*)^26,27^. Interestingly, previous brain related studies have also found some of our top DE lncRNAs, *LBX2-AS1*^28^ and *LUCAT1*^29,30^ as significantly co-expressed with other mRNAs expressed in brain (Fig. 1c). Most of the top DE PCGs where previously known to be involved in functions related to synaptic transmissions (*SHANK1*)^31^, brain abnormalities (*FOXG1*), immune response and cell-signaling (*MSN* and *CARD6*)^32,33^ (Fig. 1d). Thus, comparison using transcriptome sequencing identified most important and functionally relevant transcripts related to CCMs pathogenesis.

### Expression status of known CCM genes in patients

Though CCMs patients are characterized based on genomic mutations that occur in *KRIT1*/*CCM1*, *CCM2* and *PDCD10*/*CCM3* genes^6,34^, there was no significant change in mRNA expression patterns of these PCGs compared to other top differentially expressed lncRNA and PCGs genes (Fig. 1e). These three CCM genes are known to be strongly expressed in neural cells of brain, cerebellum and spinal cord during embryonic development and postnatal brain development^35^. But in CCMs disease context, these genes are expressed at very low level, because the mutations (two nucleotide substitutions) in *CCM1*/*KRIT1* gene causes *CCM1*/*KRIT1* mRNA to decay due to abnormal splicing and premature termination codon (PTC)^36^. Hence, there is a possibility that mRNA expression of CCM genes may be reduced by these mutations from corresponding genes^37^ (Fig. 1e). There are also possibilities of other factors that can influence the expression of these CCMs genes. These observations show the importance of investigating transcription profiles of CCMs patients in addition to genomic alterations or aberrations. Therefore, studying expression patterns of lncRNAs in CCMs may provide us additional routes for non-invasive therapies.

### Differentially expressed transcripts at CCM susceptible genomic locus

Previous studies have uncovered an association between tissue specific somatic mutations or chromosomal aberrations on transcriptional landscapes^38–41^. To characterize the downstream transcriptional effects of CCM-specific genomic aberrations, we scanned the genomic loci previously known for CCMs susceptibility and their effects on global transcriptional changes (Fig. 1f). Interestingly, we found 59 DE lncRNAs and 163 DE PCGs that map to three CCM mutated loci (CCM1: 7q11-q22, CCM2: 7p15-13 and CCM3: 3q25.2-q27): 21 DE lncRNAs and 71 DE PCGs on CCM1 locus; 21 DE lncRNAs and 41 DE PCGs on CCM2 locus; 17 DE lncRNAs and 51 DE PCGs on CCM3 locus (Fig. 1f and Supplementary Data 2). CCM locus DE lncRNAs such as *DLX6-AS1* (CCM1 locus) and *SOX2-OT* (CCM3 locus) are known to be associated with brain function and disease related to central nervous system. In addition to that, lncRNA *HOXA-AS2* (CCM2 locus) regulates malignant glioma and vasculogenic mimicry formation^42^.

### CCM associated lncRNA-mRNA co-expression analysis

**Figure 2:**
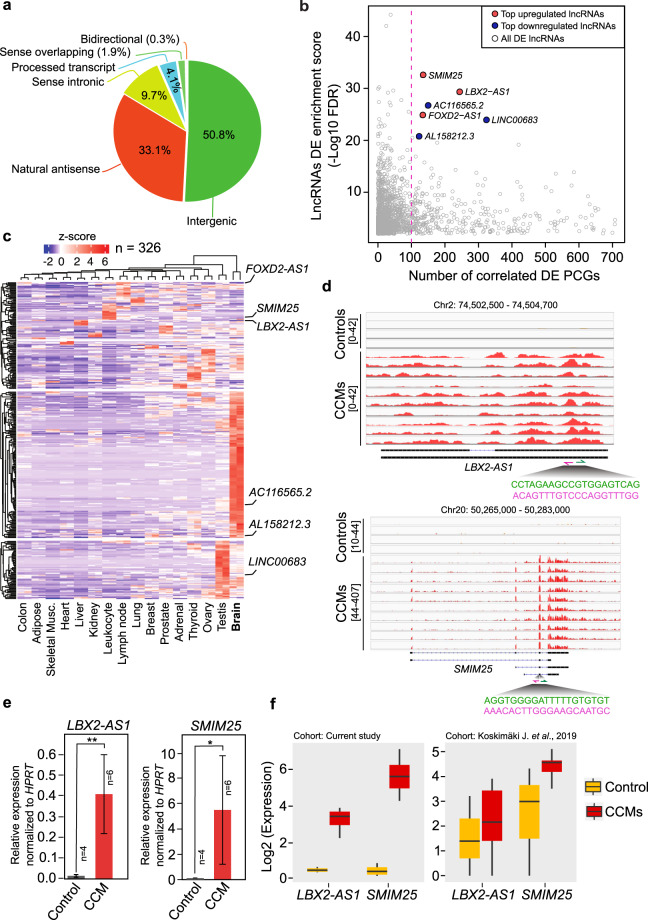
LncRNA-mRNA co-expression analysis and top lncRNA signatures. (**a**) Pie-chart with percentage of different classes of lncRNAs differentially expressed (DE) between CCMs and control group. (**b**) Scatterplot shows DE lncRNAs and its number of correlated or co-expressed DE PCGs. Left side of the pink dotted line denotes DE lncRNAs (n = 326) having more than 100 correlated DE PCGs. (**c**) Heatmap with expression status of top correlated DE lncRNAs (n = 326) in 16 normal tissues samples from human bodyMap dataset (each tissue contains 2 replicates). (**d**) IGV browser with RNA-seq read distribution of top two lncRNAs (*LBX2-AS1* and *SMIM25*) in CCM patients and control samples. Nucleotide sequences for forward and reverse primers (pink) and location of primer sequences used for qRT-PCR validations are highlighted. (**e**) qRT-PCR validation of top two DE lncRNAs. qRT-PCR graphs are presented as mean ± standard error of the mean. (**f**) Boxplots showing expression status of *LBX2-AS1* and *SMIM25* in current cohort (CCMs n = 10, controls n = 4) and cohort from Koskimäki J. *et al*. (CCMs n = 5, controls n = 3). *P*-values for qRT-PCR experiments are calculated using two-tailed student t-test. *p-value < 0.05 and **p-value < 0.01.

Since focus of our study was to investigate lncRNAs associated with CCMs pathogenesis, we observed that major DE lncRNAs belong to intergenic and natural antisense class of lncRNA (Fig. 2a). LncRNAs are also known to regulate the expression of other mRNAs via multiple mechanisms^43,44^. Therefore, comparison of the expression patterns of DE lncRNAs with PCGs was made to construct lncRNA-mRNA co-expression network. To achieve this, expression correlation analysis was performed to find significantly correlated lncRNA-mRNA pairs having Spearman coefficient above 0.9 and with correlation p-value < 0.05. This resulted in 122,112 lncRNA-mRNA significantly correlated pairs containing 1,838 DE lncRNAs and 4,379 DE PCGs. Among 1,838 DE lncRNAs, 326 lncRNAs were significantly correlated with more than 100 DE PCGs individually (Fig. 2b and Supplementary Data 1). Previous studies have shown that the expression of lncRNAs to be more tissue and cell type specific compared to PCGs. RNA-seq samples of 16 normal tissue types from human bodyMap 2.0 dataset revealed expression of these lncRNAs (n = 326) having more specificity towards brain tissue. As expected, the CCMs up-regulated DE lncRNAs showed lower expression and CCMs down-regulated DE lncRNAs showed higher expression in normal brain (Fig. 2c). Among these 326 lncRNAs, *MEG3*, *MIAT* and *SENCR* were already implicated in angiogenesis and vascular disease (angio-lncRNAs)^45^.

### Validation of *LBX2-AS1* and *SMIM25* lncRNAs

LncRNAs *LBX2-AS1* and *SMIM25*/*LINC01272* were top differentially expressed lncRNAs with more than 100 co-expressed PCGs and up-regulated in CCMs compared to control group (Fig. 2d). Further validation using RT-qPCR was performed on *LBX2-AS1* and *SMIM25* lncRNAs. Expression patterns of lncRNAs from RNA-seq analysis was successfully validated using qRT-PCR. In agreement with the findings from RNA-sequencing data, the expression of both *LBX2-AS1* and *SMIM25* transcripts were significantly up-regulated in qRT-PCR analysis. *LBX2-AS1* and *SMIM25* expressions were normalized to HPRT housekeeping gene and the normalized values were compared between control and CCMs groups (Fig. 2e). Expression status of these validated lncRNAs was also verified by analyzing RNA-seq data from a recent study from Koskimäki J *et al*. with cohorts of 5 CCM patients and 3 normal control samples^2^ (Fig. 2f). In addition to that, we have combined samples from our cohort (Controls = 4; CCMs = 10) with the samples from Koskimäki J *et al*. (Controls = 3; CCMs = 5) and performed differential expression analysis using 22 samples (7 controls vs 15 CCMs). We filtered the significant candidates using FDR/Padj <0.01 & absolute log-foldchange >1.5. Out of 190 lncRNAs from combined analysis we could find overlap of 150 lncRNAs (~79%) in our cohort’s DE analysis. Similarly, combined analysis gave 2,184 PCGs and out of which 2084 genes were overlapping with our cohort’s DE analysis. We found *LBX2-AS1* and *SMIM25* to be significantly deregulated with this combined analysis from two cohorts (Supplementary Data 1).

### CCM locus PCGs co-expressed with DE lncRNA

**Figure 3:**
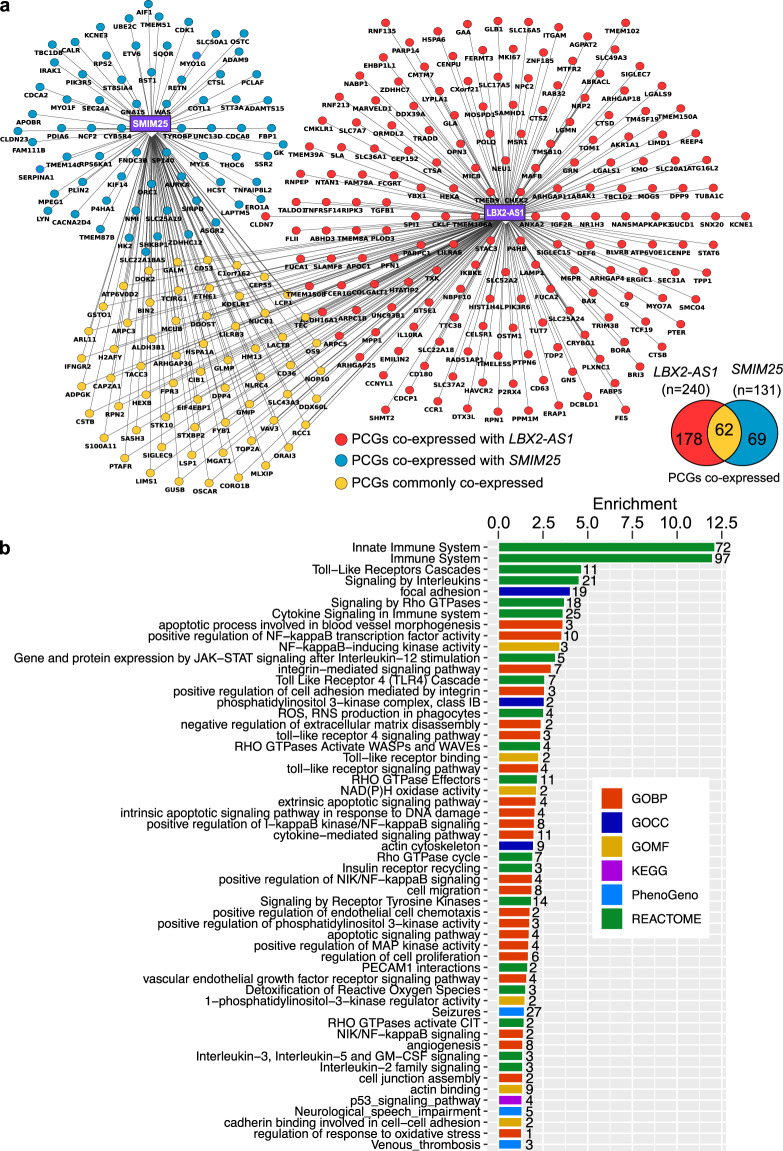
Top lncRNAs *LBX2-AS1* and *SMIM25* co-expression network and its potential co-regulatory functions. (**a**) Network shows top two lncRNAs and co-expressed DE PCGs determined by its expression correlation. Nodes and edges in the network are generated using cytoscape. Venn diagram shows common DE PCGs co-expressed between top validated lncRNAs *LBX2-AS1* and *SMIM25*. (**b**) Bar graph with enriched biological process, pathways and phenotypes derived using GeneSCF. Different color codes indicate terms derived from different database repositories. Gene ontology terms are denoted as red (Biological Process, BP), dark blue (Cellular components, CC) and yellow (Molecular Function, MF); KEGG pathways are in in purple; Reactome pathways are shown in green; and Human Phenotype Ontology terms as light blue. Enrichment in the scale denotes −log10 (*p*-value) and number above each bar represents number of genes.

There were 240 and 131 PCGs significantly correlated or co-expressed with *LBX2-AS1* and *SMIM25*/*LINC01272* respectively. Among those, 62 PCGs were commonly co-expressed with these two lncRNAs. Interestingly, several co-expressed PCGs of *LBX2-AS1* and *SMIM25* map to CCM hotspot loci CCM1: 7q11-q22, CCM2: 7p15-13 and CCM3: 3q25.2-q27 with crucial functions that have relevance to CCM pathogenesis (Fig. 3a and Supplementary Data 3). For example, *ALDH3B1*, *ARPC3*, *LIMS1*, *LSP1*, *PTAFR* and *VAV3* have been previously reported to play a role in oxidative stress, adherens junction pathway, focal adhesion and VEGF-mediated angiogenesis (Fig. 3a). Collectively, these observations suggest that the lncRNAs and their co-expressed PCGs from CCM hotspot loci could serve as potential novel biomarkers and therapeutic targets in the treatment of CCM.

### Co-expression analysis revealed functional lncRNA related to CCM pathogenesis

In order to identify functional lncRNAs in CCMs, the biological functions were predicted using GeneSCF for PCG pairs having similar expression patterns as DE lncRNAs^46^ (see methods for details). GeneSCF predicted enriched terms from various databases such as gene ontology, KEGG, The Mammalian Phenotype Ontology and Reactome pathway. Previously known important biological processes and vascular signaling pathways related to CCMs such as, VEGF receptor signaling pathway, cell junction assembly, angiogenesis, NF-kB transcription activity, focal adhesion, actin cytoskeleton, PI3K complex, Rho GTPase signaling, TLR-receptor binding and NAD(P)H oxidase activity were significantly enriched for the differentially co-expressed PCGs^47–49^ (p < 0.05) (Fig. 3b and Supplementary Data 4).

## Discussion

The advancement of high-throughput technologies such as, next generation sequencing has facilitated the identification and characterization of lncRNAs in several diseases^50^. Despite extensive studies showing the potential role of lncRNAs in cerebrovascular pathologies^17,18,51^ their identification in CCMs disease remains unexplored.

Herein applying state-of-the-art RNA-seq approach we intent to profile lncRNAs signatures in CCMs disease. Our study applying comprehensive computational analysis identified several lncRNAs and protein coding genes (PCGs) differentially expressed (DE) in CCMs. Among them, top DE lncRNAs *SMIM25* and *LBX2-AS1* were having correlative expression patterns with significant number of differentially expressed (DE) PCGs. Co-expression analysis followed by gene set enrichment prediction revealed important signaling pathways that have been previously established to be pivotal in CCMs disease development and progression. We therefore, believe that our transcriptomics-wide approach might unfold uncovered functional roles of *SMIM25* and *LBX2-AS1* and their co-expressed PCGs in CCMs pathogenesis.

*SMIM25*, also known as *LINC01272*/*GCRL1* belongs to the class of long intergenic non-coding RNA and is located in the chromosome 20. Although not well characterized in humans, recent emerging evidence suggests its association with various diseases, such as lung squamous cell carcinoma (LUSC), gastric cancer and in inflammatory bowel diseases^52–55^. Consistent with our data, the expression of *SMIM25* was found to be strongly increased in inflammatory bowel diseases and gastric cancer. Additionally, Lin *et al*. showed that upregulation of *SMIM25* is associated with gastric cell proliferation and metastasis while Haberman *et al*. demonstrated a myeloid pro-inflammatory function of *SMIM25* in pediatric Crohn disease. Wang and co-authors indicated that *SMIM25* may be used as potential diagnostic biomarkers in inflammatory bowel disease. We show here through RNA-Seq approach that *SMIM25* transcript was strongly upregulated through RNA-Seq approach and its expression change was further confirmed by qRT-PCR analysis. Functional enrichment analysis revealed CCM-relevant signaling pathways such as Rho GTPase, ROS, toll-like receptor cascades, MAP3 activation and receptor tyrosine kinase targeted by *SMIM25* as highly enriched entities. Among the total *SMIM25*-specific co-expressed PCGs, we identified *FNDC3B*, *FYB1*, *AURKA*, *GMIP*, *COTL1*, *RETN*, *ARPC3*, *RPS6KA1*, *LSP1*, *VAV3*, *CD36*, *OS9*, *ETV6*, *MYO1F*, *GSTO1*, *ADAM9*, *PTAFR*, *LIMS1*, *CDK1*, *IRAK1*, *HSPA1A* and *CLDN23* to be involved in various molecular processes such as, oxidative stress, cell-cell adhesion, integrin signaling, angiogenesis, cell proliferation, MAP kinase pathway, endothelial cell dysfunction, RhoA-GTPase activity and PI3K signaling pathways that have been previously reported to be involved in CCMs disease. For example, CD36 known as cluster of differentiation 36, is an integral membrane protein which interacts with β1 integrins to mediate Thrombospondins TSP-1 mediated apoptosis by antagonizing pro-survival pathways^56^. Reduced TSP-1 expression has been reported to contribute to CCMs pathogenesis in acute Kriti inactivated brain microvascular endothelial cells (BMECs)^57^. We, therefore, believe that CD36 might have a role to play in CCM lesion and should be studied in further detail. Another crucial PCG co-expressed with *SMIM25* was identified to be glutathione S-transferase omega-1 (*GSTO1*). *GSTO1* is a pro-inflammatory molecule and is essential for the formation of reactive oxygen species^31^ through activation of the Toll-like receptor 4 (*TLR4*) cascade^58^. Recently, Tang and co-workers indicated that endothelial *TLR4* and the gut microbiome as critical stimulators of CCMs formation while pharmacological blockage of *TLR4* signaling reduced CCMs lesion burden in mice^59^. These data suggest that *GSTO1* might also be involved in CCMs lesion formation and further studies should be conducted to uncover its potential functions. A third PCG, cyclin-dependent kinase 1 (*CDK1*) was identified as a potential candidate co-expressed with *SMIM25*. *CDK1* function as a serine/threonine kinase and acts as a potent target for the bioactive ingredient, indirubin-3-monoxime (IR3mo)^60^. In a recent study, Otten and co-workers implemented a target prediction tools and identified IR3mo as a novel candidate in rescuing CCMs phenotype in zebrafish, mice and HUVEC models^61^. It would therefore be intriguing to conduct further studies and determine if *CDK1* might have a functional role in CCMs pathogenesis.

*LBX2-AS1* or ladybird homeobox 2 antisense RNA 1 belongs to antisense long non-coding RNA class and is located within the chromosome 2. Recent reports indicate its importance in gliomas, esophageal squamous cell carcinoma and lung adenocarcinoma^28,62–64^. Intriguingly, Liang and co-workers applied weighted gene co-expression network analysis (WGCNA) and showed that *LBX2-AS1* was significantly associated with focal adhesion, extracellular matrix receptor (ECM) interaction and MAPK signaling pathways (Liang *et al*., 2018). Our results showing a strong up-regulation of *LBX2-AS1* might suggest its significant role in CCMs disease. Furthermore, we also identified several PCGs such as *ARHGAP25*, *BAX*, *MKI67*, *PFN1*, *TGFβ1*, *ANXA2*, *CELSR1*, *CCR1*, *CDCP1*, *CKLF*, *CLDN7*, *EMILIN2*, *FERMT3*, *GRN*, *HEXA*, *IGF2R*, *LGALS9*, *MAPKAPK3*, *P2RX4*, *RNF213*, *TMEM106A* and *TRADD* known to be associated with various processes like, inflammation, angiogenesis, cell proliferation, Rho GTPase, β-catenin pathway, adherens junctions, apoptosis, cell adhesion, MAP kinase/Erk pathway and nitric oxide pathways. Among them, Bcl-2-associated protein, BAX, a pro-apoptotic regulator has been established to be involved in CCMs pathogenesis. Recent studies from Antognelli and co-workers demonstrated that the pro-apoptotic protein BAX was significantly increased in KRIT1^−/−^ cells, which are characterized by an elevated apoptotic rate^65^. We also identified Antigen-KI-67 (MKI67), a cell proliferation marker co-expressed with *LBX2-AS1*. *MKI67* has been reported to be strongly expressed in CCMs tissues, suggesting high proliferative index in these lesions^66,67^. Transforming growth factor beta 1 or TGF-β1, a polypeptide belonging to the transforming growth factor beta superfamily of cytokines was identified as a co-expressed PCG with *LBX2-AS1*. The role of TGF-β1 has been previously established in CCMs diseases. Maddaluno *et al*., demonstrated that the TGF-β1 signaling pathway activates endothelial to mesenchymal transition (EndMT) in CCM1-ablated endothelial cells and inhibition of the TGF-β1 signaling pathway prevented EndMT both *in vitro* and *in vivo*, subsequently reducing the size of vascular lesions in *CCM1*-deficient mice^68^. Additionally, Bravi and co-workers reported that TGF-β1 signaling is triggered downstream following β-catenin activation in *CCM3*-deficient endothelial cells both *in vitro* and *in vivo* and leads to endothelial-to-mesenchymal transition, implying TGF-β/BMP signaling as crucial regulators for CCMs disease progression^69^. Profilin 1 (*PFN1*) an actin monomer-binding protein belonging to the profilin family was identified to be a PCG co-expressed with *LBX2-AS1*. It has been previously established that profilin acts as a binding partner for the junctional multidomain protein, Afadin-6 (AF-6)^70^. Earlier Glading *et al*. have shown that KRIT-1 associates with Rap1 small GTPases and transfection of BAECs with activated Rap1 (RapV12) led to increased association of endogenous KRIT-1 with β-catenin and AF-6^71^. Contrarily, their association was strongly inhibited following expression of the Rap activity inhibitor (Rap1GAP). Further studies will discern the role of profilin in CCMs pathogenesis.

Besides, we identified 62 PCGs that were commonly co-expressed with *SMIM25* and *LBX2-AS1*. Among them, *ALDH3B1*, *ARPC3*, *LIMS1*, *LSP1*, *PTAFR* and *VAV3* have been previously validated to play crucial roles in oxidative stress^72^, adherens junction pathway^73^, focal adhesion^74^, *PECAM1*-mediated expression in endothelial cells^75^, VEGF-mediated angiogenesis^76^ and vascular endothelial cell integrity^77^. These observations in particular warrants further studies to explore their functional relevance in CCMs disease.

The Gene Ontology for biological process (GOBP), cellular components (GOCC) and molecular functions (GOMF) analysis revealed VEGF receptor signaling pathway, cell junction assembly, angiogenesis, NF-kB transcription activity, focal adhesion, actin cytoskeleton, PI3K complex, TLR-receptor binding and NAD(P)H oxidase activity as highly enriched processes in CCMs pathogenesis. The Reactome pathway analysis identified immune system, TLR-receptor cascades, signaling by interleukins, Rho GTPases signaling, cytokine signaling and reactive oxygen species^31^ as significantly enriched pathways. Among this, VEGF is one of the key factors for CCMs pathogenesis which induces endothelial proliferation (Fig. 3b)^78^. There is also evidence showing that CCM1 expression resulted in significant reduction of VEGF-induced sprout formation during endothelial cell differentiation and capillary formation in a 3D spheroidal system^79^. The authors also detected that cell migration in CCM-expressing HUVECs was significantly delayed in a Boyden chamber using VEGF as a chemoattractant. A previous study has also shown that loss of *CCM2* increased Rho GTPase activity in CCMs^80^. Consistent with this previous study our analysis also predicted Rho GTPase related pathways were enriched with CCMs up-regulated genes while there is no significant expression of *CCM2* gene (Figs. 1e and 3b). Apart from these known pathways in CCMs, it is also important to thoroughly investigate other biological pathways reported in this study to understand the importance of these DE lncRNAs. Additionally, phenotype enrichment analysis reflected terms related to CCMs symptoms such as, seizures, neurological speech impairment (focal neurologic deficit) and venous thrombosis (Fig. 3b). All this evidence suggests that CCMs differentially expressed lncRNAs with similar expression patterns as DE PCGs might co-regulate important pathways relevant to CCMs pathogenesis.

We provide here a comprehensive transcriptomic profile identifying lncRNAs and their co-expressed PCGs in human resected CCM lesions. To the best of our knowledge, this is the first high-throughput study to show their differential expression patterns in brainstem CCMs. Our results identified lncRNAs, *SMIM25* and *LBX2-AS1* and their commonly co-expressed PCGs; *ALDH3B1*, *ARPC3*, *LIMS1*, *LSP1*, *PTAFR* and *VAV3* as strong candidates in human CCMs disease. Future studies understanding their functional role in CCMs biology will open new perspectives for better therapeutic targets for disease prevention and treatment.

## Materials and Methods

### Ethical statement

All procedures performed in studies involving human participants were in accordance with the ethical standards of the responsible committee (institutional and national) and with the 1964 Helsinki declaration and its later amendments. The study protocol was approved by the local ethical committee at the Hannover Medical School, Germany (Approval Number 6960) and the Ethics Committee of University Bonn Medical Center (Approval Number 076/08 and 042/08). Written informed consent was collected from all the patients involved in this study.

### Patients and clinical data

Fresh tissue biopsies were obtained intraoperatively from 10 adult patients (CCM1-10) affected due CCMs within the brainstem and stored at −80 °C until further experiments. Normal brain tissues from 4 subjects who underwent temporal lobe epilepsy (TLE) surgery at the University Hospital Bonn, Germany served as corresponding controls. The control tissue samples were treated in an identical manner as CCMs tissues. The clinical diagnosis of the CCMs was based on MRI and histopathological characteristics as previously described^81^. Each CCMs patient manifested at least one or more clinical episodes of seizures, hemorrhage and focal neurological deficits. The patient clinical characteristics are described in Tables 1 and 2. For research involving participant or patient under the age of 18 years (including donors of tissue samples), informed consent was taken from the parent.

### RNA isolation

Tissue sections were stored in at least 10 volumes of RNAlater-ICE (AM7030; Ambion) at −80 °C before total RNA extraction. For RNA extraction, brain tissue specimens were cut off and further slit to smaller pieces. These pieces were transferred into vials containing ceramic beads (Precellys system, CK14L; Peqlab) and 700 µl of RLT lysis buffer from RNeasy Micro Kit (74004; Qiagen) including 1% of beta-mercaptoethanol. Homogenization was performed by use of the Precellys 24 Homogenizer (Peqlab) with 2–4 pulses of 5 sec at 6000 rpm. Total RNA was extracted with RNeasy Micro Kit according to the company’s recommendations including an on-column DNase-I digestion step (5 minutes at RT). Total RNA was finally eluted with 14 µl of RNase free water. RNA samples were quantified and purity was determined with the Nanodrop ND-1000 spectrophotometer (Peqlab). RNA integrity was determined with an Agilent Bioanalyzer 2100 using the RNA 6000-Nano assay (5067-1511; Agilent Technologies). RNA Integrity Numbers (RINs) of samples used for RNA-Sequencing experiments ranged from 7.0 to 8.4 with an average RIN of 7.6 for the CCMs samples and 7.5 for the control samples.

### RNA library preparation, quality control, and quantification

200 ng of total RNA per sample were utilized as input for rRNA depletion procedure with ‘NEBNext® rRNA Depletion Kit (Human/Mouse/Rat), 96 rxns’ (E6310X; New England Biolabs) followed by stranded cDNA library generation using ‘NEBNext® Ultra Directional RNA Library Prep Kit for Illumina’ (E7420L; New England Biolabs). All steps were performed as recommended in user manualE7420 (Version 6.0_08-2015; NEB) except that all reactions were downscaled to 2/3 of initial volumes. Furthermore, one additional purification step was introduced at the end of the standard procedure, using 1x ‘Agencourt® AMPure® XP Beads’ (#A63881; Beckman Coulter, Inc.).

cDNA libraries were barcoded by single indexing approach, using ‘NEBNext Multiplex Oligos for Illumina – Set 1’ (Index Primer 2, 3, 4, 5, 6, 7, and 12). All generated cDNA libraries were amplified by 11 cycles of final PCR. Fragment length distribution of individual libraries was monitored using ‘Bioanalyzer High Sensitivity DNA Assay’ (5067-4626; Agilent Technologies). Quantification of libraries was performed by use of the ‘Qubit® dsDNA HS Assay Kit’ (Q32854; ThermoFisher Scientific).

### Library denaturation and sequencing run

Equal molar amounts of seven individually barcoded libraries were pooled. This library pool was denatured with NaOH and was finally diluted to 1.5pM according to the Denature and Dilute Libraries Guide (Document #15048776 v02; Illumina). 1.3 ml of denatured pool was loaded on an Illumina NextSeq. 550 sequencer using a High Output Flowcell for 75 bp single read sequencing (#FC-404-2005; Illumina).

### Raw data processing and quality control

BCL files were converted to FASTQ files using bcl2fastq Conversion Software version 2.16.0.10 (Illumina). The FASTQ files were adapter and quality trimmed using Trim Galore (version 0.4.1) with default settings as described in the User Guide except for the setting of the quality cutoff (−q/−quality) which was set to a Phred score of 15. Trim Galore used Cutadapt (version 1.9.1) as subroutine. Quality control of FASTQ files was performed by FastQC (version 0.11.4) before and after trimming.

### Transcriptome mapping and assembly

After trimming, FASTQ files were mapped against a reference human genome hg38 with the splice-aware aligner STAR (version 2.5.0c)^82^ to generate BAM files. The BAM files were built in a 2-pass Mapping (–twopassMode Basic) and were finally sorted (–outSAMtype BAM SortedByCoordinate). All other setting have been left as default as described in the manual. Homo sapiens sequence and annotation data (UCSC, build hg38) used from illumina’s iGenome site (http://support.illumina.com/sequencing/sequencing_software/igenome.html).

### Differential expression and lncRNA-mRNA co-expression analysis

Obtained aligned files (BAM format) were subjected to quantification using featureCounts from Subread package^83^. The reads were quantified for Ensembl transcript annotation release GRCh38.93 (corresponds to genome hg38)^84^ with featureCounts parameters ‘–minOverlap 10-Q30-s2-ignoreDup -J’. Generated matrix file from read quantification was then used for differential expression (DE) analysis. DE analysis was performed using Bioconductor package DESeq2^85^ with contrast of normal and CCMs groups. Transcripts (lncRNAs and protein coding genes) having corrected p-value or Padj <0.01 (FDR) and absolute log-fold change greater than 1.5 were considered to be significantly differentially expressed (DE). For calculating the coding potential of DE lncRNAs and PCGs we started by extracting nucleotide sequence (FASTA) of the DE transcripts using BioMart from Ensembl. Obtained sequences (FASTA) are used as an input for CPC2 (Coding potential calculator v2) tool to predict coding probability of the transcripts^86^.

Expression patterns of DE lncRNAs were compared against DE protein coding genes (PCGs) to look for expression correlation. Significant lncRNA-mRNA correlated pairs were calculated using Spearman correlation coefficient by considering R value above 0.9 and with p-value < 0.05. The co-expression network linking lncRNA-mRNA pairs were constructed using Cytoscape^87^.

### Gene set enrichment analysis

PCGs co-expressed (significantly correlated) with top validated lncRNAs was used for gene set enrichment analysis. These PCGs were used to predict phenotype (Human Phenotype Ontology), gene ontology (GOBP, GOCC, GOMF) and molecular pathways (Reactome) using GeneSCF tool^46^. Significant terms were considered, if having p-value < 0.05.

### Processing RNA-seq public dataset from multi-tissues

Human BodyMap transcriptome sequencing data (E-MTAB-513) was processed by aligning with STAR^82^, quantified with featureCounts^83^ and normalized as Transcript Per Million (TPM). Expression of CCMs differentially expressed and top correlated lncRNAs were tested in 2 × 16 (two replicates each) normal tissue samples including brain. The heatmap from Fig. 2c is plotted by calculating z-score or standard score from the normalized TPM values to check specificity of DE lncRNAs in different tissues.

### Processing CCM patient RNA-seq public dataset

RNA-seq samples of 5 CCM patients and 3 healthy samples from neurovascular units (NVU) were downloaded from ‘GSE123968’. Samples were processed and treated in similar way as cohort from current study and also similar to Human BodyMap RNA-seq samples. We combined RNA-seq samples from our cohort (Controls = 4; CCMs = 10) with this Koskimäki J *et al*. 2019 cohort (Controls = 3; CCMs = 5). Differential expression analysis was performed using Bioconductor package DESeq2 with contrast of normal (7 controls) and CCMs groups (15 CCMs). Transcripts (lncRNAs and protein coding genes) having corrected p-value or Padj <0.01 (FDR) and absolute log-fold change greater than 1.5 were considered to be significantly differentially expressed (DE).

### Technical validation of lncRNA expression by qRT-PCR

In order to validate the reliability of RNA-sequencing data, we randomly selected *SMIM25* and *LBX2-AS1*, the two most strongly upregulated lncRNAs for further analysis. SYBR green-based quantitative polymerase chain reaction (qRT-PCR) assay was used for this purpose. Briefly, DNase1-treated total RNA of 2 µg was reverse transcribed to cDNA using a High-Capacity cDNA Reverse Transcription kit (Applied Biosystems). The expression analysis of the *SMIM25* and *LBX2-AS1* primer was carried out using PowerUp™ SYBR™ Green Master Mix (Applied Biosystems) on a StepOnePlus Real-time PCR (Applied Biosystems). The specific forward and reverse primers for the lncRNAs were designed using Primer 5.0. Thermal cycling conditions consisted of an initial denaturing step at 95 °C for 10 min and then 40 cycles. The relative expression and fold change for the *SMIM25* and *LBX2-AS1* lncRNA was normalized by HPRT using the 2^−ΔΔCt^ method and each qRT-PCR assay was repeated in triplicates.

### Statistical analysis

All experimental graphs from qRT-PCR are presented as mean ± standard error of the mean and a *p* value less than 0.05 value was considered statistically significant. The p-values of qRT-PCR was derived using two-tailed student t-test. From RNA-seq, the significantly differentially expressed lncRNAs and protein coding genes were determined by DESeq2^85^ and filtered using adjusted *p* value and log-fold change values.

### Ethics declarations

Written informed consent was collected from all the patients involved in this study.

## Supplementary information

<b>Supplementary Information</b>

<b>Supplementary data 1</b>

<b>Supplementary data 2</b>

<b>Supplementary data 3</b>

<b>Supplementary data 4</b>

## Data Availability

The RNA-seq data used in this publication can be accessed from GEO repository with accession GSE137596 (https://www.ncbi.nlm.nih.gov/geo/query/acc.cgi?acc=GSE137596).
