# Supplementary material for: Transcriptome-wide Profiling of Cerebral Cavernous Malformations Patients Reveal Important Long noncoding RNA molecular signatures
Source: Sci Rep. 2019 Dec 3;9:18203. doi: 10.1038/s41598-019-54845-0 (PMC6890746; doi:10.1038/s41598-019-54845-0)
Supplement: Supplementary file 1 — Supplementary Information [file 41598_2019_54845_MOESM1_ESM.docx]

**Supplementary Information**

**Transcriptome-wide Profiling of Cerebral Cavernous Malformations Patients Reveal Important Long noncoding RNA molecular signatures**

Santhilal Subhash^2, ǂ^, Norman Kalmbach^3^, Florian Wegner^4^, Susanne Petri^4^, Torsten Glomb^5^, Oliver Dittrich-Breiholz^5^, Caiquan Huang^1^, Kiran Kumar Bali^6^, Wolfram S Kunz^7^, Amir Samii^1^, Helmut Bertalanffy^1^, Chandrasekhar Kanduri^2, *^, Souvik Kar^1, ǂ, *^

^1^International Neuroscience Institute, Rudolf-Pichlmayr-Strasse 4, D-30625, Hannover, Germany

^2^Department of Medical Biochemistry and Cell Biology, Institute of Biomedicine, Sahlgrenska Academy, University of Gothenburg, Gothenburg 40530, Sweden

^3^Neopep Pharma GmbH & Co. KG, Feodor Lynen Strasse 31, 30625, Hannover, Germany (present address)

^4^Department of Neurology, Hannover Medical School, Hannover, Germany

^5^Research Core Unit Genomics, Hannover Medical School, Hannover, Germany

^6^Department for Experimental Pain Research, Center of Biomedicine and Medical Technology Mannheim (CBTM), Medical Faculty Mannheim, Heidelberg University, Mannheim, Germany

^7^Institute of Experimental Epileptology and Cognition Research and Department of Epileptology, Life and Brain Center, University Hospital Bonn, Sigmund-Freud-Strasse 25, D-53105, Bonn, Germany

^ǂ^ These authors contributed equally to this work.

* These are co-corresponding authors

**Address for Correspondence to:**

1. Souvik Kar, Ph.D,

E-mail: [kar@ini-hannover.de](mailto:kar@ini-hannover.de)

1. Prof. Chandrasekhar Kanduri

E-mail: [kanduri.chandrasekhar@gu.se](mailto:kanduri.chandrasekhar@gu.se)

**Supplementary Datasheets**

**Supplementary data 1.** List of significantly differentially expressed lncRNAs and PCGs between CCM patients and control samples (XLSX).

**Supplementary data 2.** Differentially expressed transcripts (lncRNA and PCGs) found on CCM susceptibility locus (XLSX).

**Supplementary data 3.** Co-expressed DE PCGs with top two lncRNAs, *LBX2-AS1* and *SMIM25* (XLSX).

**Supplementary data 4.** Gene functional enrichment analysis of *LBX2-AS1* and *SMIM25* co-expressed PCGs (XLSX).
